# Interactive effects of polygenic risk and cognitive subtype on brain morphology in schizophrenia spectrum and bipolar disorders

**DOI:** 10.1007/s00406-022-01450-4

**Published:** 2022-07-06

**Authors:** Yann Quidé, Oliver J. Watkeys, Leah Girshkin, Manreena Kaur, Vaughan J. Carr, Murray J. Cairns, Melissa J. Green

**Affiliations:** 1 School of Clinical Medicine, Discipline of Psychiatry and Mental Health, University of New South Wales (UNSW), Sydney, NSW Australia; 2 Neuroscience Research Australia, Randwick, NSW Australia; 3 Department of Psychiatry, Monash University, Clayton, VIC Australia; 4 School of Biomedical Sciences and Pharmacy, University of Newcastle, Callaghan, NSW Australia; 5 Centre for Brain and Mental Health Research, University of Newcastle, Callaghan, NSW Australia; 6 Hunter Medical Research Institute, New Lambton Heights, NSW Australia

**Keywords:** Cross-disorder, Genetic risk, Schizophrenia, Bipolar disorder, Cognition, Grey matter volume

## Abstract

Grey matter volume (GMV) may be associated with polygenic risk for schizophrenia (PRS-SZ) and severe cognitive deficits in people with schizophrenia, schizoaffective disorder (collectively SSD), and bipolar disorder (BD). This study examined the interactive effects of PRS-SZ and cognitive subtypes of SSD and BD in relation to GMV. Two-step cluster analysis was performed on 146 clinical cases (69 SSD and 77 BD) assessed on eight cognitive domains (verbal and visual memory, executive function, processing speed, visual processing, language ability, working memory, and planning). Among them, 55 BD, 51 SSD, and 58 healthy controls (HC), contributed to focal analyses of the relationships between cognitive subtypes, PRS-SZ and their interaction on GMV. Two distinct cognitive subtypes were evident among the combined sample of cases: a ‘cognitive deficit’ group (CD; *N* = 31, 20SSD/11BD) showed severe impairment across all cognitive indices, and a ‘cognitively spared’ (CS; *N* = 75; 31SSD/44BD) group showed intermediate cognitive performance that was significantly worse than the HC group but better than the CD subgroup. A cognitive subgroup-by-PRS-SZ interaction was significantly associated with GMV in the left precentral gyrus. Moderation analyses revealed a significant negative relationship between PRS-SZ and GMV in the CD group only. At low and average (but not high) PRS-SZ, larger precentral GMV was evident in the CD group compared to both CS and HC groups, and in the CS group compared to HCs. This study provides evidence for a relationship between regional GMV changes and PRS-SZ in psychosis spectrum cases with cognitive deficits, but not in cases cognitively spared.

**Supplementary Information:**

The online version contains supplementary material available at 10.1007/s00406-022-01450-4.

## Introduction

Considerable evidence indicates that schizophrenia spectrum disorders (SSD; including schizophrenia, schizoaffective disorder) and bipolar disorder (BD) share clinical features [1], genetic risk factors [2, 3], brain aberrations [4], and cognitive deficits [5, 6]. Individuals with these disorders also show substantial inter-individual variation in symptom profiles and illness course, such that the utility of studying both within- and cross-disorder subtypes defined by cognitive performance [7–9] and/or biological markers (i.e., biotypes) [10] has gained traction in the past decade, within the Research Domain Criteria (RDoC) framework [11]. Moving beyond traditional comparison of heterogeneous diagnostic groups to study relations between biological risk factors (e.g., genetics) and endophenotypes (e.g., cognitive deficits, brain morphology) shared among some individuals with these diagnoses provides one means of addressing the heterogeneity of these factors within conditions. The present study used this approach to examine relationships between polygenic risk for schizophrenia and grey matter volume among cross-disorder subtypes of patients with SSD and BD defined by their cognitive profile.

With neuropsychological deficits well recognised as an important determinant of functional outcome in psychosis [12–14]. These deficits are associated with shared brain abnormalities across the schizophrenia and mood disorder spectrum [15], likely highlighting similar cognitive profiles, clinical characteristics, grey matter reduction, across diagnoses. Verbal memory, sustained attention, spatial ability, processing speed, executive function, and language [16, 17], are largely impacted, persist over time (irrespective of illness phases), and run in families [18, 19] of cases with SSD and BD. Studies have demonstrated the utility of using cognitive features of illness to determine subgroups within, or across, SSD and BD [20]. Despite mounting evidence for a subtype with relatively spared (or ‘near-normal’) functioning in both BD and SSD, estimates of the proportion of clinical groups with ‘near-normal cognition’, versus a ‘severe cognitive deficit’ group, vary considerably between studies and appear to be dependent on methodological differences between studies [20].

Schizophrenia spectrum disorders are associated with reduced grey matter volume and cortical thickness across the whole brain [15, 21], with disruption of white matter pathways [22, 23]. Recent studies have examined these brain-based phenotypes in relation to polygenic risk for schizophrenia, representing the degree to which multiple sites of genetic variation contribute to risk for schizophrenia: this can be summarised using ‘polygenic risk scores’ (PRS), calculated as the sum of alleles associated with a particular trait, weighted by their respective effect sizes [24]. Increased PRS for schizophrenia (PRS-SZ) is associated with reduced total brain volume and white matter in schizophrenia cases and/or healthy subjects [25, 26]. However, a recent review [27] suggests that the current evidence for associations between PRS-SZ and brain structure is inconclusive.

With shared polygenic risk for SSD and BD now well-established [24, 28–30], it is plausible that any association between PRS-SZ and brain-based phenotypes may extend to cases with BD who show more severe cognitive deficits. Regardless of the number of subgroups delineated in these analyses, there is some consensus in reporting a subgroup with severe cognitive deficits that show reduced fronto-temporal grey matter volume and cortical thickness relative to various reference groups showing various degrees of relatively spared cognition [15, 31, 32]. Recent studies have begun to examine cognitive- and brain-based phenotypes in relation to polygenic risk for schizophrenia (PRS-SZ), with increasing PRS-SZ being associated with more pronounced cognitive deficits [33, 34]. However, a recent review highlights inconsistent associations between PRS-SZ and brain morphology [27], and no studies of cognitive-based subtypes have investigated the potential effects of PRS-SZ on brain structure. In this study, we aimed to estimate the interactive effects of PRS-SZ and cognitive status [with cognitive deficits (CD) or cognitively spared (CS), determined for cross-disorder SSD/BD groups] on whole-brain grey matter volume. We expected that higher PRS-SZ would be associated with reduced fronto-temporal grey matter volume in cognitive-deficit cases.

## Methods

All procedures involving human subjects/patients were approved by the UNSW Human Research Ethics (HC12384), the South East Sydney and Illawarra Area Health Service (HREC 09/081) and St Vincent’s Hospital committees (HREC/10/SVH/9).

### Participants

Two-hundred-and-forty-five individuals were initially recruited into the study: 166 met the International Classification of Diseases (ICD-10) criteria for a primary psychotic or affective disorder [35] including 80 with a diagnosis of either schizophrenia (*n* = 50), schizoaffective disorder (*n* = 29), or delusional disorder (*n* = 1), referred to collectively as schizophrenia spectrum disorders (SSD; *n* = 80), and 86 who met criteria for a diagnosis of bipolar disorder (BD); the remaining 79 individuals were healthy controls (HC) with no history of an ICD-10 axis-I disorder as determined by the Mini International Neuropsychiatric Interview [36] and no history of psychosis in first-degree biological relatives. Following data screening and quality control procedures applied to cognitive, magnetic resonance imaging, and genetic data for the purpose of this study, there were 146 clinical participants (69 SSD, 77 BD) included in cognitive subtyping analyses. The demographic, clinical, and cognitive characteristics of these diagnostic groups and healthy participants are summarised in Supplementary Tables 1 and 2, and illustrated in Supplementary Fig. 1. Focal analyses of PRS and brain morphometry were conducted for a slightly smaller sample comprising 164 participants (51 SSD, 55 BD and 58 HC) who had all biological data available for analysis.

Participants were recruited from local area health services, the Australian Schizophrenia Research Bank [37], the Sydney Bipolar Disorder Clinic [38], and advertisements in the local community. General exclusion criteria included an inability to communicate sufficiently in English, a current neurological disorder, a diagnosis of substance abuse or dependence in the past 6 months; and/or having been treated with electroconvulsive therapy in the previous 6 months.

### Materials

#### Clinical assessment

ICD-10 diagnoses were confirmed using the Operational Criteria Checklist for Psychotic Illness and Affective Illness (OPCRIT) algorithm [39] applied to interviewer ratings on the Diagnostic Interview for Psychosis (DIP) [40]. The DIP was also used to confirm a lifetime history of psychotic symptoms, defined as the lifetime occurrence of hallucinations and/or delusions during at least one illness episode [41]. Current symptom severity was assessed in cases using the Positive and Negative Syndrome Scale (PANSS) [42]. In addition, all participants completed the State-Trait Anxiety Inventory (STAI) [43], the Montgomery–Åsberg Depression Rating Scale (MADRS) [44] and the Young Mania Rating Scale (YMRS) [45]. All participants additionally completed the Wechsler Abbreviated Scale of Intelligence (WASI) [46] and Wechsler Test of Adult Reading (WTAR) [47]. Data on antipsychotic and antidepressant medications were collected via self-report and transformed to chlorpromazine (CPZ) and imipramine (IMI) equivalent dosages, respectively [48, 49]. Use of mood stabilizers, including carbamazepine, lithium, lamotrigine or valproate, was also recorded.

#### Cognitive assessments

Cognitive performance was assessed by a comprehensive battery of standardised tests that spanned seven domains, following the method described by Reichenberg et al. [17]. The cognitive domains included were: verbal memory, visual memory, executive function, processing speed, visual processing, working memory, and planning. Scores on a number of tests were reversed so that lower scores were indicative of poorer cognitive performance (see below).

*Verbal memory* was measured using the List Learning, List Recall, Story Memory, and Story Recall subtests from the Repeatable Battery for the Assessment of Neuropsychological Status (RBANS) [50].

*Visual memory* was measured by the RBANS Figure Recall subtest and ‘Within-search Errors’ measure of the Cambridge Neuropsychological Test Automated Battery (CANTAB) [51] Spatial Working Memory (SWM) test.

*Executive function* was measured using the time on part B of the Trail-Making Test (TMT reverse scored) [52], the total score on the Controlled Oral Word Association Test (COWAT) [53], the RBANS Semantic Fluency subtest score, and the ‘Total Errors (adjusted; reverse-scored)’ and ‘Stages Completed’ measures of the CANTAB Intra/Extra-dimensional (IED) test. Domain scores were calculated for participants with valid data on at least two of the four tasks.

*Processing speed* was measured using the time on part A of the Trail-Making Test (reverse-scored), the Digit Symbol Coding subtest score from the Wechsler Adult Intelligence Scale-Revised (WAIS-R) [54] and the RBANS Coding subtask. Domain scores were calculated for participants with valid data on at least two of these three tasks.

*Visual processing* was measured by the WASI Matrix Reasoning subtest score, and the RBANS Figure Copy and Line Orientation subtest scores.

*Working memory* was measured using the WAIS-R Digit Span and Letter-Number Sequencing (LNS) subtest scores, and the CANTAB SWM ‘Between-search Errors’ measure (reverse-scored).

*Planning* was measured using several indices derived from the CANTAB Stockings of Cambridge (SOC) task, including ‘Problems solved in minimum moves’, ‘Mean moves for 5-move problems’ (reverse-scored), ‘Mean initial thinking time for a 5-move problem’ and ‘Mean subsequent thinking time for a 5-move problem’ measures of, as well as the SWM ‘Strategy’ score (reverse-scored).

#### Genetic data processing and polygenic risk score calculation

*Genotyping and QC procedure* The Infinium PsychArray-24 BeadChip (Illumina; San Diego, CA, USA) was used in accordance with the manufacturer’s instructions to conduct genotyping on DNA quantification via optical density. The Illumina HiScan and Infinium iScan Control Software (ICS v3.3.28) with the PsychChip_15048346 manifest applied, were used to determine the fluorescence intensity of the beads in each array. Normalised intensity data were derived using Illumina’s GenomeStudio v2011.1 software with the Genotype Module v1.9.4. The genomic quality control (QC) procedure commenced with the removal of single nucleotide polymorphisms (SNPs) demonstrating a call rate < 95%. Participants evidencing: (1) a call rate < 98%, (2) evidence of inbreeding (genome-wide heterozygosity |FHET|> 0.2), or (3) discrepant sex data between genotype and phenotype, were then removed from the sample. Finally, SNPs that showed: (1) a call rate < 98%, (2) differential missing > 0.02, (3) a Hardy–Weinberg equilibrium (HWE) *p* value < 1e−6 in cases, (4) a HWE *p* value < 1e−8 in controls, (5) were invariant, or (6) had a small minor allele frequency (MAF) < 0.01 [55], were removed from the dataset. Gene imputation was carried out using the Michigan Imputation Server [56] for all autosomal chromosomes using the 1000G Phase 3 v5 reference panel and Eagle v2.3 phasing. Upon completion of imputation, the resulting output was subject to further QC procedures in line with guidelines recommended by the Enhancing NeuroImaging Genetics through Meta-Analyses (ENIGMA) consortium [57]. Following these protocols 7,836,062 SNPs were retained.

*PRS calculation* Polygenic risk scores for schizophrenia (PRS-SZ) were calculated using PRSice v2.3.2 [58], using effect sizes from a meta-analysis of schizophrenia Genome-Wide Association Studies (GWAS) in European and South-East Asian cohorts [59]. Of the 202 subjects (132 cases and 70 controls) with genetic data available, 182 were of European or South-East Asian ancestry and passed QC protocols (61 HC, 63 BD, and 58 SZ). Participants of other genetic ancestry were not included in any analysis including PRS-SZ. A series of PRSs were calculated at *p* value thresholds (*p*T) between 0.01 and 0.5 to determine the optimal *p*T which explained the greatest amount of variance in schizophrenia clinical status. Whilst schizophrenia PRSs were calculated for all participants of European and South-East Asian ancestry, only the HC and SZ participants were used in determining the optimal *p*T. Results suggested that the PRS-SZ calculated at 1.4005e−03 explained the greatest amount of variance in case–control status (*p* = 0.004, *R*^2^ = 0.101, nSNPs = 7593), and this was adopted for subsequent analyses. Following principal component analysis, two components were significantly associated with ethnicity (*p* < 0.05) and were included as covariates in all subsequent analyses including PRS-SZ.

#### Structural MRI data acquisition and pre-processing

High-resolution T1-weighted anatomical scans (MPRAGE) were obtained on a Philips 3 T Achieva TX scanner (Philips Healthcare, Best, The Netherlands) housed at Neuroscience Research Australia (Randwick, NSW, Australia) with a 32-channel head coil for each participant (TR 8.9 ms, TE 4.1 ms, field of view 240 mm, matrix 268 × 268, 200 sagittal slices, slice thickness 0.9 mm, no gap). All scans successfully passed a quality assessment protocol in which a radiologist reviewed all scans, and an additional visual inspection for gross artefacts and movements (presence of excessive ringing that would not allow identification of two adjacent brain regions) was followed by an automated quality control using the Computational Anatomy Toolbox (CAT12.6, v1433; http://dbm.neuro.uni-jena.de/cat/index.html) for SPM12 (v7487; Wellcome Trust Centre for Neuroimaging, London, UK; http://www.fil.ion.ucl.ac.uk/spm) in Matlab r2014b (Mathworks Inc., Sherborn, MA, USA). Structural scans were pre-processed using CAT12 default routine for voxel-based morphometry (VBM). The VBM processing pipeline included segmentation of the scans into grey matter (GM), white matter (WM) and cerebrospinal fluid (CSF) segments that were normalized to a standard Montreal Neurological Institute (MNI152) template using the SPM's “Diffeomorphic Anatomic Registration Through Exponentiated Lie” algebra normalization (DARTEL) [60]. In addition, partial volume effects [61], hidden Markov Random Field model [62] and adaptive maximum a posteriori estimations [63] were applied to the segmentation. Normalized images were additionally modulated with the Jacobian determinants of the deformation parameters. Following these steps, an additional quality control on sample homogeneity was performed to ensure there were no outlier scans with a Mahalanobis distance between mean correlations and weighted overall image quality significantly higher than the other scans. Grey matter images were smoothed with a 8 mm full width at half maximum (FWHM) Gaussian kernel for second-level (group comparison) VBM analyses. Finally, total intracranial (TIV), total GM, total WM and total CSF volumes were also extracted for each participant. Grey matter images were smoothed with a 8 mm full width at half maximum (FWHM) Gaussian kernel for second-level (group comparison) voxel-based morphology (VBM) analyses.

### Statistical analysis

All analyses were conducted using SPSS (version 25; IBM SPSS Statistics). Prior to cluster analysis of cognitive domains (conducted only for data from SSD and BD participants), a Mahalanobis distance test (*p* < 0.001) accounting for the influence of one test on another within the same domain, was used to identify outliers for cognitive performance within the HC group. These outliers were removed, and all test scores for these outliers within the domain were treated as missing. Following outlier removal, clinical cases scores on each cognitive task were converted to *z*-scores (relative to HC performance) and averaged to create *z*-scores for each domain. Clinical cases missing a value for one or more of the domains (BD = 9, SSD = 11) were excluded from all further analyses. *Z*-scores were re-calculated for all eligible participants following this procedure and converted to *t* scores for use in all subsequent analyses.

### Cluster analysis and characteristics of derived cognitive subtypes

For 146 clinical participants with valid data on all cognitive domains, *t* scores for each cognitive domain were entered into a two-step agglomerative hierarchical cluster analysis, with the log-likelihood distance measure used as the similarity criterion to determine cluster membership; solutions for 2 classes, 3 classes, and 4 classes were compared on Schwarz's Bayesian Information Criterion (BIC) to determine the optimal number of clusters. A series of one-way analyses of covariance (ANCOVAs) including age and sex as covariates, were used to examine differences between the 2 classes (Cognitive Deficit, CD; Cognitively Spared, CS) derived from the optimal cluster solution on cognitive domain scores.

### Effects of PRS and cognitive group, and their interaction, on brain imaging measures

A series of whole-brain multiple linear regressions were used to determine the associations between PRS-SZ, group (cognitive-subtype or clinical diagnosis, examined separately), and their potential interaction, on whole-brain GMV (VBM analyses); age, sex, TIV and the indices of ethnicity stratification were included as covariates. Statistical significance was set at a stringent voxel-wise family-wise error (FWE) corrected significance threshold of *p*FWE = 0.025 (two-tailed), with a minimum cluster size of 30 contiguous voxels. Significant group-by-PRS-SZ interactions were followed up with formal moderation analyses using the PROCESS macro (v3.4) [64] for SPSS. Two sets of moderation analyses were performed with the extracted raw signal at the cluster peak as the dependent variable: in the first moderation analysis, the effects of group (independent variable) were tested at three levels of the PRS-SZ (moderator): at 1 standard deviation (SD) below to the average PRS-SZ (low PRS-SZ), at average PRS-SZ, and at 1 SD above to the average PRS-SZ (high PRS-SZ) [65]. In the second moderation analysis, the effects of PRS-SZ (independent variable) were tested for each group (moderator). In all analyses, age, sex, TIV and the indices of ethnicity stratification were entered as covariates. Sensitivity analysis was performed in clinical cases only, accounting for chlorpromazine (CPZ) and imipramine (IMI) equivalent dosages and mood stabiliser usage to assess whether any of our findings were attributable to extraneous effects of medication. The Davidson–McKinnon correction (HC3) was used to account for potential issues related to heteroskedasticity [66]. Statistical significance was set at a threshold of *p* < 0.05.

## Results

### Cognitive subtypes

Cluster analyses of seven cognitive domains (*t* scores) produced a two-cluster solution (BIC = 5263.53) that had fair cohesion and separation (silhouette measure = 0.42) and was a better fit to the data than a model with 3 clusters (ratio of BIC change = − 0.08) or 4 clusters (ratio of BIC change = − 0.29). The first cluster, referred to as the cognitively spared (CS) subtype, comprised 68% of the combined SSD and BD participants (*n* = 99); a second cluster, referred to as a cognitive-deficit (CD) subtype, comprised 32% of participants (*n* = 47). The proportion of cases from traditional diagnostic groups distributed between cognitive subtypes was significantly different: the CS subtype comprised 61% BD cases (*n* = 61) and 39% SSD cases (*n* = 38), while the CD subtype comprised 66% SSD cases (*n* = 31) and 34% BD (*n* = 16). However, a lifetime history of psychosis was not significantly different between cognitive subtypes (CS *n* = 94 of 99 cases; CD *n* = 44 of 47 cases).

**Fig. 1:**
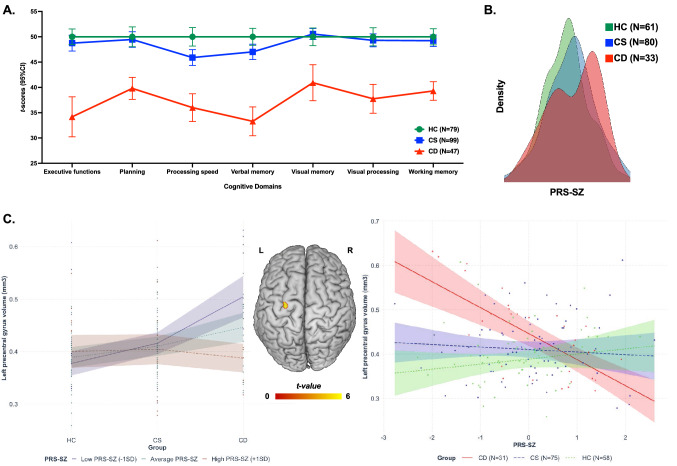
Cognitive performances, distribution of polygenic risk score for schizophrenia (PRS-SZ) and brain features associated with group-by-PRS-SZ interaction. **A** Performance on all studied domains for the cognitive subtypes. The cognitively spared group (CS, green squares) performed at a similar level compared to the healthy control group (HC, blue spheres), but the group with cognitive deficits (CD, red triangles) performed lower than both the HC and CD groups. **B** Distribution of the PRS-SZ among the cognitive subtypes. **C** Results from the moderation analyses following significant group-by-PRS-SZ interaction. (Left panel) When PRS-SZ was entered as the moderator, the CD group had larger precentral GMV than both the CS and HC groups, with the CS group showing larger precentral GMV than the HC group, only for those with low (diamond, steal dashed line) or average (circles, steal dotted line), but not high (brown star, dash-dotted line) levels of PRS-SZ. (Right panel) When groups were entered as moderators, decreased grey matter volume in the left precentral gyrus was associated with higher PRS-SZ in the CD group (red triangles), but not in CS (green squares) or in HC groups (blue circles). L: left; R: right; the colour-bar represents *t* statistics

Demographic information and cognitive performance on each of the cognitive domains used in the cluster analyses are presented for the HC, CS and CD subtypes (compared to each other, and to HCs) in Table 1, and illustrated in Fig. 1A. The CS subtype showed little difference in cognitive performance on all cognitive domains except for processing speed and verbal memory compared to HCs, while the CD group had significantly impaired cognitive performance relative to both HCs and CS cases, except for domains of planning and visual processing for which the performance of CS and CD subtypes did not differ. The CD group was marginally older than the HC group and had less years of education than both the CS and HC groups. The CD group also had lower premorbid IQ relative to the CS and lower current IQ relative to both the CS and HC groups. In addition, the CS group had less years of education than the HC group.

**Table 1:** Demographic and cognitive characteristics of cluster-derived subgroups and healthy controls

	HC		CS		CD		Statistics				Pairwise comparisons (*p* values)		
	*N*	Mean (SD)	*N*	Mean (SD)	*N*	Mean (SD)	*F*/*χ*^2^	*df*	*p* value	$\eta_{{\text{p}}}^{2}$/*φ*	HC vs CS	HC vs CD	CS vs CD
*Demographics*													
Age	79	36.03 (11.50)	99	38.12 (11.90)	47	41.76 (12.37)	**3.432**	2222	**0.034**	**0.030**	0.731	**0.028**	0.256
Years of Education^a^	79	16.82 (2.58)	98	15.69 (2.84)	47	14.29 (2.50)	**12.344**	2219	** < 0.001**	**0.101**	**0.018**	** < 0.001**	**0.018**
WTAR^a^	79	108.72 (14.53)	98	107.43 (11.79)	47	91.83 (15.61)	**23.138**	2219	** < 0.001**	**0.174**	1.000	** < 0.001**	** < 0.001**
WASI^b^	56	117.2 (12.28)	80	114.56 (10.43)	32	97 (12.17)	**35.350**	2164	** < 0.001**	**0.301**	0.650	** < 0.001**	** < 0.001**
Sex (M/F)	43/36		41/58		27/20		4.543	2	0.103	0.142			
Diagnosis (SSD/BD)	–	–	38/61		31/16		**9.722**	**1**	**0.002**	**0.258**			
Lifetime psychosis (DIP)	–	–	94		44		0.109	1	0.712	0.027			
*Cognitive domains*													
Executive functions^a^	73	50.00 (6.65)	99	48.74 (7.87)	47	34.18 (13.44)	42.584	2214	** < 0.001**	**0.285**	1.000	** < 0.001**	** < 0.001**
Planning^a^	56	50.00 (7.32)	99	49.46 (7.70)	47	39.80 (7.44)	29.586	2197	** < 0.001**	**0.231**	1.000	** < 0.001**	** < 0.001**
Processing speed^a^	79	50.00 (8.14)	99	45.90 (7.86)	47	36.00 (9.34)	38.379	2220	** < 0.001**	**0.259**	**0.002**	** < 0.001**	** < 0.001**
Verbal memory^a^	78	50.00 (7.45)	99	47.02 (7.57)	47	33.30 (9.71)	63.848	2219	** < 0.001**	**0.368**	**0.031**	** < 0.001**	** < 0.001**
Visual memory^a^	72	50.00 (7.42)	99	50.54 (5.49)	47	40.93 (12.10)	24.009	2213	** < 0.001**	**0.184**	1.000	** < 0.001**	** < 0.001**
Visual processing^a^	67	50.00 (7.37)	99	49.29 (6.42)	47	37.75 (9.72)	49.142	2208	** < 0.001**	**0.321**	1.000	** < 0.001**	** < 0.001**
Working memory^a^	74	50.00 (6.92)	99	49.23 (5.69)	47	39.30 (6.24)	48.985	2215	** < 0.001**	**0.313**	1.000	** < 0.001**	** < 0.001**

Significant group differences are in bold (*p* < 0.05)

*HC* healthy controls, *CS* cognitively spared clinical cases, *CD* clinical cases with cognitive deficits, *N* number of subjects, *SD* standard deviation, *df* degrees of freedom, *WTAR* Wechsler Test of Adult Reading, *WASI* Wechsler Abbreviated Scale of Intelligence, *M/F* male/female, *SSD* schizophrenia spectrum disorder, *BD* bipolar disorder, *DIP* Diagnositic Interview for Psychosis

^a^Age and sex were included as covariates

^b^Sex was included as covariate

### Descriptive statistics for the cluster-derived cognitive subtypes

#### Clinical characteristics

Clinical characteristics of the cluster-derived cognitive subtypes are presented in Table 2. State anxiety (measured with the STAI) was higher in both the CD (*p* < 0.001) and CS (*p* < 0.001) groups relative to HCs, but there were no differences between the CD and CS groups in state anxiety levels (*p* = 1.000). Among the clinical cases, the CD subtype showed higher levels of negative, general and total, but not positive symptoms on the PANSS, compared to the CS subtype, but there were no significant differences between the subtypes in terms of depressive (MADRS) and manic (YMRS) symptoms. There were no differences between CD and CS subtypes on antidepressant and antipsychotic medication dosages, but the use of mood stabilisers was significantly greater in the CS group (*n* = 58 of 99 cases), compared to the CD group (*n* = 15 of 47 cases), likely due to the high proportion of BD cases.

**Table 2:** Clinical characteristics, brain morphometry and polygenic risk scores of cluster-derived cognitive subtypes and health participants

	HC			CS		CD		Statistic			
	*N*		Mean (SD)	*N*	Mean (SD)	*N*	Mean (SD)	*F/t/χ*^2^	*df*	*p* value	$\eta_{{\text{p}}}^{2}$/*d*
MADRS^a^				99	9.77 (9.11)	46	10.52 (9.13)	0.151	1141	0.698	0.001
YMRS^a^				99	6.37 (7.69)	46	7.74 (7.33)	0.777	1141	0.380	0.005
PANSS Positive^a^				99	12.21 (7.34)	47	14.45 (6.09)	1.665	1142	0.199	0.012
PANSS Negative^a^				99	11.42 (4.77)	47	15.15 (6.83)	**11.048**	**1142**	**0.001**	**0.072**
PANSS General^a^				99	24.92 (7.32)	47	28.79 (9.37)	**4.567**	**1142**	**0.034**	**0.031**
PANSS Total^a^				99	48.56 (14.73)	47	58.38 (18.19)	**8.078**	**1142**	**0.005**	**0.054**
Age of onset (years)				91	23.85 (9.01)	42	25.57 (9.49)	1.010	131	0.314	0.186
Length of illness (years)				98	15.49 (9.39)	46	18.35 (11.42)	1.585	142	0.115	0.274
Antidepressant dosage (mg)^a,b^				28	120.17 (132)	17	110.77 (108.42)	0.239	141	0.628	0.006
Antipsychotic dosage (mg)^a,c^				64	553.24 (1092.88)	35	516.1 (700.49)	0.103	195	0.749	0.001
Mood stabilizer use, N Yes				58		15		**9.068**	**1**	**0.003**	**0.249**
STAI (State anxiety)	79	28.86 (8.58)		98	38.62 (12.2)	47	37.62 (11.48)	**19.540**	**2219**	** < 0.001**	**0.151**
Total GMV in mm^3 d^	58	637.94 (65.86)		75	623.94 (54.73)	31	623.77 (53.84)	1.674	6312	0.127	0.031
Total WMV in mm^3 d^	58	511.54 (67.37)		75	501.18 (55.25)	31	504.38 (45.46)				
Total CSF in mm^3 d^	58	261.13 (59.12)		75	276.85 (57.83)	31	271.73 (70.82)				
PRS-SZ^e^	61	− 26.48 × 10^–4^ (2.43 × 10^–4^)		80	− 25.95 × 10^–4^ (2.71 × 10^–4^)	33	25.69 × 10^–4^ (2.76 × 10^–4^)	**3.639**	**2167**	**0.028**	**0.042**
PRS-SZ range (Min–Max)	61	− 32.17 × 10^–4^ to − 20.08 × 10^–4^		80	− 33.42 × 10^–4^ to − 19.29 × 10^–4^	33	− 31.37 × 10^–4^ to − 21.09 × 10^–4^				

Significant group differences are in bold (*p* < 0.05)

*HC* healthy controls, *CS* cognitively spared clinical cases, *CD* clinical cases with cognitive deficits, *N* number of subjects, *SD* standard deviation, *STAI* state and trait anxiety inventory, *MADRS* Montgomery and Åsberg depression rating scale, *YMRS* Young mania rating scale, *PANSS* positive and negative symptoms scale, *GMV* grey matter volume, *WMV* white matter volume, *CSF* cerebrospinal fluid, *PRS-SZ* polygenic risk score for schizophrenia

^a^Age and sex were included as covariates

^b^Antidepressant dosage = imipramine equivalent

^c^Antipsychotic dosage = chlorpromazine equivalent

^d^MANCOVA included age, sex and TIV as covariates

^e^ANCOVA included age, sex and the indices of ethnicity stratification as covariates

#### Polygenic risk scores among cognitive subtypes

Descriptive statistics of PRS-SZ among the cognitive subtypes are presented in Table 2, and the distribution of PRS-SZ among the groups is illustrated in Fig. 1B. Univariate analyses of covariance (ANCOVAs) (controlling for age, sex and the first two principal components of ethnicity stratification) were used to examine differences between PRS-SZ among groups: there was a significant main effect of cognitive subtype on PRS-SZ, and post-hoc tests showed higher PRS-SZ in the CS (*p* = 0.023) but not the CD (*p* = 0.566) group when each was compared to the HC group; PRS-SZ was not significantly different between CS and CD groups (*p* = 1.000). In addition, to rule-out any multi-collinearity effect, an ANCOVA confirmed there were no significant direct PRS-SZ differences between the CD and CS groups only [*F*(1,107) = 0.797, *p* = 0.374]. Comparable statistics for analyses conducted with diagnosis groups are presented Supplementary Table 1 and Supplementary Fig. 2, showing that PRS-SZ was higher in the SSD group compared to both the HC (*p* < 0.001) and BD groups (*p* = 0.003), and no significant PRS-SZ difference between HC and BD (*p* = 1.000).

#### Structural brain differences among cognitive subtypes

There was no significant effect of cognitive subtype (HC, CS, CD) on the total GMV, WMV, and CSF masks; Table 2 presents the descriptive and summary statistics from multivariate analysis of covariance (MANCOVA) to determine the main effects (*p* < 0.05), followed by within-group univariate ANCOVAs where appropriate (Bonferroni-corrected *p* < 0.017); age, sex and total intracranial volume (TIV) were entered as covariates of non-interest. In addition, a one-way ANCOVA (with age, sex and TIV included as covariates) was used to determine the voxel-wise differences in whole-brain GMV among the cognitive groups (HC, CS, CD). An absolute masking threshold of 0.2 was set to avoid the inclusion of non-GM voxels in all whole-brain voxel-wise analyses. There were no significant between-groups whole-brain GMV differences. Comparable analyses showed no differences in total GMV, WMV, CSF masks (see Supplementary Table 2) or whole-brain VBM among participant groups (HC, BD, SSD).

### Whole brain GMV regression analyses

In the absence of any significant direct effects of PRS-SZ or group on GMV assessed using whole-brain VBM analysis, the cognitive group-by-PRS interaction was significantly negatively associated with GMV in the left precentral gyrus (peak MNI coordinates [− 28, − 10.64], *k* = 47, *t*_155_ = − 5.25, *z* = 5.03, *p*FWE = 0.010; see Fig. 1C and Table 3 for statistics associated with this cluster). After extraction of the raw GMV at the cluster peak, the model testing the direct effects of PRS-SZ, group or their interaction on GMV was significant [*R*^2^ = 0.443, *F*(10,153) = 13.273, *p* < 0.001]. Within this model, the direct effect of group and the interaction effect were significant [*R*^2^ change = 0.117, *F*(2,153) = 16.791, *p* < 0.001], but not the direct effect of PRS-SZ.

**Table 3:** Results from two moderation analyses testing either PRS or cognitive group as the moderator of the other’s association with GMV at the peak voxel in the significant left precentral gyrus cluster (MNI coordinates [− 28, − 10.64])

	*b*	se	*t*-statistic	*p* value	LLCI	ULCI
*Direct association with PRS-SZ*						
	43.540	36.917	1.179	0.240	− 29.392	116.472
*Direct association with cognitive groups*						
**CS vs HC**	**0.021**	**0.011**	**1.992**	**0.048**	** < 0.001**	**0.043**
**CD vs HC**	**0.057**	**0.013**	**4.412**	** < 0.001**	**0.032**	**0.083**
**CS vs CD**	**0.036**	**0.013**	**2.694**	**0.008**	**0.010**	**0.062**
*Interactions*						
PRS-SZ × group (CS vs HC)	− 65.099	45.957	− 1.417	0.159	− 155.890	25.693
**PRS-SZ × group (CD vs HC)**	− **266.649**	**47.057**	− **5.667**	** < 0.001**	− **359.615**	− **173.682**
**PRS-SZ × group (CS vs CD)**	**201.550**	**49.134**	**4.102**	** < 0.001**	**104.481**	**298.619**
*PRS-SZ as moderator*						
**Low PRS-SZ CS vs HC**	**0.039**	**0.015**	**2.628**	**0.010**	**0.010**	**0.068**
**Low PRS-SZ CD vs HC**	**0.128**	**0.021**	**6.091**	** < 0.001**	**0.087**	**0.170**
**Low PRS-SZ CS vs CD**	− **0.090**	**0.021**	− **4.260**	** < 0.001**	− **0.131**	− **0.048**
**Average PRS-SZ CS vs HC**	**0.021**	**0.011**	**1.992**	**0.048**	** < 0.001**	**0.043**
**Average PRS-SZ CD vs HC**	**0.057**	**0.013**	**4.412**	** < 0.001**	**0.032**	**0.083**
**Average PRS-SZ CS vs CD**	− **0.036**	**0.013**	− **2.694**	**0.008**	− **0.062**	− **0.010**
High PRS-SZ CS vs HC	0.004	0.018	0.233	0.816	− 0.031	0.039
High PRS-SZ CD vs HC	− 0.013	0.014	− 0.931	0.354	− 0.042	0.015
High PRS-SZ CS vs CD	0.018	0.016	1.109	0.269	− 0.014	0.049
*Group as moderator*						
HC	43.540	36.917	1.179	0.240	− 29.392	116.472
CS	− 21.559	32.702	− 0.659	0.511	− 86.165	43.047
**CD**	− **223.109**	**37.752**	− **5.910**	** < 0.001**	− **297.691**	− **148.526**

Significant associations are in bold

*PRS-SZ* polygenic risk score for schizophrenia, *HC* healthy controls, *CS* cognitively spared cases with BD or SSD, *CD* cases with BD or SSD with cognitive deficits, *se* standard error, *LLCI* lower limit 95% confidence interval, *ULCI* upper limit 95% confidence interval

The first moderation analysis testing PRS-SZ as the moderator of associations between cognitive subtype and GMV revealed that cases from the CD group at low and average, but not high PRS-SZ, showed significantly greater GMV in the left precentral cluster than both the HC and CS groups. Similarly, cases from the CS group with low and average, but not high PRS-SZ had significantly greater GMV in the left precentral cluster than the HC group.

The second moderation analysis testing ‘cognitive subgroup’ as the potential moderator of the relationships between PRS-SZ and GMV in the left precentral cluster revealed a negative relationship between PRS-SZ and grey matter in this cluster for the CD group only; the associations between PRS-SZ and GMV were not significant for the CS or the HC group (see Table 3).

Sensitivity analysis in SSD and BD cases demonstrated that all effects remained statistically significant when accounting for CPZ and IMI equivalent dosages, as well as mood stabiliser usage within the model (see Supplementary Table 3).

Comparable whole-brain regression analyses exploring the interaction between PRS-SZ and traditional clinical diagnostic groups revealed no significant main effects or interactions on whole-brain VBM.

## Discussion

This study delineated two distinct cognitive subtypes among clinical cases with schizophrenia spectrum (SSD) and bipolar disorders (BD)—one with severe cognitive deficit (CD) across all assessed domains, and the other subtype characterised by relatively spared cognitive performance (CS; comparable to healthy controls)—for investigation of differences in neuroanatomical features in the context of individual levels of polygenic risk for schizophrenia (PRS-SZ). We revealed a significant interaction between PRS-SZ and cognitive subtype on grey matter volume in the left precentral gyrus: formal moderation analyses revealed that decreased GMV in this region was significantly associated with higher PRS-SZ scores within the cognitive-deficit (CD) group only (i.e., when subgroup was tested as the moderator). This was revealed in the context of no direct effects of either cognitive subgroups or PRS-SZ on grey matter volume (GMV). Sensitivity analyses suggested that none of the significant effects observed could be attributed to use of psychotropic medications.

However, when the PRS-SZ was examined as the potential moderator of associations between cognitive subgroup and GMV, the CD group showed larger precentral GMV compared to both the cognitively spared (CS) and healthy control (HC) groups at low or average, but not high PRS-SZ levels. This finding suggests that excessive neural growth in the precentral gyrus is associated with lower genetic risk loading for SZ; this is not implausible, with consideration of previous findings of excessive synaptic pruning linked to schizophrenia-associated genetic variation [67]. Thus, increased genetic risk for schizophrenia might somehow attenuate abnormal precentral growth. While this remains speculative, it is also possible that exposure to other environmental risk factors affects neurodevelopment among these cases with extreme cognitive-deficit (e.g., socioeconomic deprivation, stress/trauma, substance use) [68, 69]. Alternatively, this finding may be related to heterogeneity of genetic risk in this relatively small sample (discussed further below). Overall, the present findings are inconsistent with previous studies reporting a lack of association between PRS-SZ and brain morphology in schizophrenia, when cognitive profiles were not considered [27].

The two cognitive subgroups identified in this cohort of mixed SSD and BD cases included one group characterised by cognitive-deficit that accounted for approximately a third (32%) of the clinical sample, in line with previous solutions which have all delineated a group with global cognitive impairment, regardless of the number of classes [20]. Previous cross-disorder studies using slightly different methodology (e.g. different cognitive tests or analytic techniques) have produced three [9] or four [70] subtypes, whereas studies that have derived subtypes within groups of SSD or BD cases separately revealed two [71], three [72], and five cognitive subtypes [20]. As with other studies, cases with cognitive deficits delineated in this sample were characterised by more severe negative and general symptoms relative to CS cases [71]. Given previous evidence for less cognitive impairment in BD groups as a whole [17, 73], it is not surprising that the distribution of clinical cases among cognitive subtypes shows a higher prevalence of near-normal neuropsychological functioning in BD compared to SSD; that is, the CS cluster was comprised 62% BD patients, while the CD cluster was made up of 34% BD patients. However, unlike other studies in BD [72, 74] or mixed SSD/BD samples [75], we found no evidence for the existence of a subgroup distinguished by selective cognitive impairments (on processing speed, attention, working memory and verbal learning). This may, at least in part, explain the lack of GMV differences between the CS and CD groups regardless of PRS-SZ; previous studies of three cognitive subtypes have reported larger precentral GMV in cognitively impaired relative to selectively impaired cases [31].

Interestingly, the CS group, but not the CD group, had higher average polygenic risk scores relative to the HC group, while average polygenic risk scores were not significantly different among the cognitive subgroups. This finding is somewhat surprising, and inconsistent with previous findings of higher PRS-SZ in association with cognitive deficits [33, 34]. However, a larger PRS-SZ is not unexpected in BD cases relative to HCs [29], and here BD cases comprised a high proportion of the CS group. This finding may therefore simply be a reflection of heterogeneity in polygenic risk for schizophrenia in this sample. Further investigations in larger cohorts that maximise the distribution of cognitive deficits are warranted. It may also be interesting to examine these associations using a PRS derived in association with human cognitive performance rather than that associated with schizophrenia.

Limitations of this study include the relatively small sample size for genetic analyses, compared to studies of national and international consortia [5]. Although sensitivity analyses showed no impact of medication on the results, our mixed cohort has a limited capacity to account for the potential effect of differential medication use (antidepressant, antipsychotics, mood stabilizers), and their length of use, on other regional grey matter changes [76, 77]. The potential variability over the course of illness, which may impact the consistency of assessments over different phases of illness, may also have influenced our results. Finally, the finding that the PRS-SZ was higher in the group of patients with CS (which comprised a relatively lower percentage of patients with SSD as compared to the CD group) is puzzling and worth mentioning as a potential limitation related to the small study sample; this finding may reflect limited (or indeed extreme) heterogeneity of polygenic risk scores in this small sample, and/or the low number of CD cases relative to CS.

In summary, this study examined the association between polygenic risks for schizophrenia and neuroanatomical features of two distinct cognitive subtypes among clinical cases with schizophrenia spectrum and bipolar disorders—one group showed severe cognitive deficits across all assessment domains, and the other showed relatively spared cognitive abilities. Higher PRS-SZ scores were associated with decreasing volume of the precentral gyrus in the cognitive-deficit subgroup only; greater volume of this region was associated with lower PRS-SZ scores in all cases regardless of cognitive profile. The findings call for further research of shared genetic risk for intermediate phenotypes across diagnostic categories of schizophrenia, schizoaffective disorder, or bipolar disorder.

## Supplementary Information

Below is the link to the electronic supplementary material.

Supplementary file1 (DOCX 497 kb)

## Data Availability

Not applicable.
