# Supplementary material for: Interactive effects of polygenic risk and cognitive subtype on brain morphology in schizophrenia spectrum and bipolar disorders
Source: Eur Arch Psychiatry Clin Neurosci. 2022 Jul 6;272(7):1205–18. doi: 10.1007/s00406-022-01450-4 (PMC9508053; doi:10.1007/s00406-022-01450-4)
Supplement: Supplementary file 1 — Supplementary file1 (DOCX 497 kb) [file 406_2022_1450_MOESM1_ESM.docx]

**Interactive effects of polygenic risk and cognitive subtype on brain morphology in schizophrenia spectrum and bipolar disorders**

**- Supplementary Material -**

Yann Quidé^1,2^, Oliver J. Watkeys^1,2^, Leah Girshkin^1,2^, Manreena Kaur^1,2^, Vaughan J. Carr^1,2,3^, Murray J. Cairns^4,5,6^, Melissa J. Green*^1,2^

^1^ School of Psychiatry, University of New South Wales (UNSW), Sydney, NSW, Australia

^2^ Neuroscience Research Australia, Randwick, NSW, Australia

^3^ Department of Psychiatry, Monash University, Clayton, VIC, Australia

^4^ School of Biomedical Sciences and Pharmacy, University of Newcastle, Callaghan, NSW, Australia

^5^ Centre for Brain and Mental Health Research, University of Newcastle, Callaghan, NSW, Australia

^6^ Hunter Medical Research Institute, New Lambton Heights, NSW, Australia

*Corresponding author

Professor Melissa J. Green

Room 101, AGSM Building, Botany Street, Kensington, NSW, 2052, Australia

Phone: +61 (0)2 8382 1584

Fax: +61 (0)2 8382 1402

Email: melissa.green@unsw.edu.au

| **Supplementary Table 1. Demographic and cognitive characteristics of traditional diagnostic and healthy participant groups** | | | | | | | | | | | | | |
| --- | --- | --- | --- | --- | --- | --- | --- | --- | --- | --- | --- | --- | --- |
|  | HC | | BD | | SSD | | Statistics | | | | Pairwise comparisons (*p*-values) | | |
|  | N | Mean (SD) | N | Mean (SD) | N | Mean (SD) | *F / χ^2^* | *df* | *p*-value | *η_p_^2^* / *φ* | HC vs BD | HC vs SSD | BD vs SSD |
| **Demographics** |  |  |  |  |  |  |  |  |  |  |  |  |  |
| Age | 79 | 36.03 (11.50) | 77 | 36.85 (12.39) | 69 | 42.02 (11.31) | **5.50** | 2,222 | **0.005** | **0.047** | 1.000 | **0.007** | **0.026** |
| Years of Education^ | 79 | 16.82 (2.58) | 77 | 15.94 (2.83) | 68 | 14.44 (2.57) | **13.552** | 2,219 | **<0.001** | **0.110** | 0.115 | **<0.001** | **0.006** |
| WTAR^ | 79 | 108.72 (14.53) | 77 | 104.06 (15.70) | 68 | 100.46 (14.05) | **4.261** | 2,219 | **0.015** | **0.037** | 0.134 | **0.016** | 1.000 |
| WASI^ | 56 | 117.2 (12.28) | 59 | 111.47 (12.36) | 53 | 107.40 (14.48) | **7.891** | 2,163 | **0.001** | **0.088** | 0.072 | **<0.001** | 0.311 |
| Sex (M/F) | 43/36 |  | 28/49 |  | 40/29 |  | **8.063** | **2** | **0.018** | **0.189** |  |  |  |
| Lifetime psychosis | - | - | 71 |  | 67 |  | 1.683 | 1 | 0.281 | 0.107 |  |  |  |
|  |  |  |  |  |  |  |  |  |  |  |  |  |  |
| **Cognitive domains** |  |  |  |  |  |  |  |  |  |  |  |  |  |
| Executive functions^ | 73 | 50.00 (6.65) | 77 | 48.24 (8.33) | 69 | 39.82 (13.65) | 16.310 | 2,214 | **<0.001** | **0.132** | 0.674 | **<0.001** | **<0.001** |
| Planning^ | 56 | 50.00 (7.32) | 77 | 47.41 (8.79) | 69 | 45.16 (8.80) | 3.945 | 2,197 | **0.021** | **0.039** | 0.231 | **0.017** | 0.817 |
| Processing speed^ | 79 | 50.00 (8.14) | 77 | 44.78 (9.22) | 69 | 40.41 (9.42) | 18.354 | 2,220 | **<0.001** | **0.143** | **<0.001** | **<0.001** | **0.186** |
| Verbal memory^ | 78 | 50.00 (7.45) | 77 | 45.63 (9.07) | 69 | 39.23 (10.99) | 20.171 | 2,219 | **<0.001** | **0.156** | **0.002** | **<0.001** | **0.010** |
| Visual memory^ | 72 | 50.00 (7.42) | 77 | 49.26 (5.32) | 69 | 45.41 (12.09) | 3.833 | 2,213 | **0.023** | **0.035** | 1.000 | **0.035** | 0.071 |
| Visual processing^ | 67 | 50.00 (7.37) | 77 | 46.42 (8.60) | 69 | 44.63 (10.07) | 6.436 | 2,208 | **0.002** | **0.058** | 0.118 | **0.001** | 0.353 |
| Working memory^ | 74 | 50.00 (6.92) | 77 | 47.93 (6.29) | 69 | 43.91 (8.13) | 12.418 | 2,215 | **<0.001** | **0.104** | 0.288 | **<0.001** | **0.003** |
| HC: healthy controls; BD: cases diagnosed with bipolar disorder; SSD: cases diagnosed with schizophrenia spectrum disorders; SD: standard deviation; df: degrees of freedom  ^ Age and sex were included as covariates  Significant group differences are in bold. | | | | | | | | | | | | | |

| **Supplementary Table 2. Clinical characteristics, brain morphometry and polygenic risk scores of traditional diagnostic and healthy participant groups** | | | | | | | | | | | |  |
| --- | --- | --- | --- | --- | --- | --- | --- | --- | --- | --- | --- | --- |
|  | HC | | | BD | | SSD | | Statistic | | | | |
|  | N | | Mean (SD) | N | Mean (SD) | N | Mean (SD) | *F/t/χ^2^* | df | p-value | *η_p_^2^/d/φ* | |
| MADRS^ |  | | | 76 | 9.87 (9.00) | 69 | 10.16 (9.26) | 0.005 | 1,141 | 0.945 | <0.001 | |
| YMRS^ |  | | | 76 | 6.41 (6.53) | 69 | 7.25 (8.62) | 0.218 | 1,141 | 0.642 | 0.002 | |
| PANSS Positive^ |  | | | 77 | 11.29 (6.92) | 69 | 14.77 (6.72) | **5.524** | **1,142** | **0.020** | **0.037** | |
| PANSS Negative^ |  | | | 77 | 10.55 (4.56) | 69 | 14.94 )6.10) | **18.138** | **1,142** | **<0.001** | **0.113** | |
| PANSS General^ |  | | | 77 | 25.16 (7.18) | 69 | 27.25 (9.15) | **0.329** | **1,142** | **0.567** | **0.002** | |
| PANSS Total^ |  | | | 77 | 47.03 (13.29) | 69 | 56.96 (18.21) | **7.841** | **1,142** | **0.006** | **0.052** | |
| Age of onset |  | | | 73 | 23.90 (9.95) | 60 | 24.98 (8.14) | 0.675 | 131 | 0.501 | 0.119 | |
| Length of illness |  | | | 76 | 14.38 (9.79) | 68 | 18.68 (10.09) | **2.594** | **142** | **0.010** | **0.433** | |
| Antidepressant dosage^a^^ |  | | | 19 | 86.25 (80.74) | 26 | 138.81 (143.08) | 3.122 | 1,41 | 0.085 | 0.071 | |
| Antipsychotic dosage^b^^ |  | | | 40 | 380.05 (670.60) | 59 | 648.63 (1119.60) | 1.169 | 1,95 | 0.282 | 0.012 | |
| Mood stabilizer use |  |  | | 60 |  | 13 |  | **50.810** | **1** | **<0.001** | **0.59** | |
| STAI^ | 79 | 28.86 (8.58) | | 76 | 39.32 (12.19) | 69 | 37.17 (11.64) | **20.210** | **2,219** | **<0.001** | **0.156** | |
| Total GMV in mm^3 #^ | 58 | 637.94 (65.86) | | 55 | 618.72 (51.78) | 51 | 629.47 (56.71) | 0.892 | 6,312 | 0.501 | 0.017 | |
| Total WMV in mm^3 #^ | 58 | 511.54 (67.37) | | 55 | 494.30 (51.55) | 51 | 510.54 (52.45) |  |  |  |  |  |
| Total CSF in mm^3 #^ | 58 | 261.13 (59.12) | | 55 | 264.56 (56.97) | 51 | 286.99 (64.80) |  |  |  |  |  |
| PRS-SZ ^$^ | 61 | -26.48x10^-4^ (2.43x10^-4^) | | 58 | -26.66 x10^-4^ (2.74x10^-4^) | 55 | 25.05x10^-4^ (2.46x10^-4^) | **9.101** | **2,167** | **<0.001** | **0.098** | |
| PRS-SZ range (Min-Max) | 61 | -32.17x10^-4^ – -20.08x10^-4^ | | 58 | -33.42x10^-4^ – -19.29x10^-4^ | 55 | -31.37x10^-4^– -21.09 x10^-4^ |  |  |  |  | |
| HC: healthy controls; BD: cases diagnosed with bipolar disorder; SSD: cases diagnosed with schizophrenia spectrum disorders; N: number of subjects; M: mean; SD: standard deviation; M/F: male/female; WTAR: Wechsler test for adult reading; WASI: Wechsler abbreviated scale of intelligence; STAI: state and trait anxiety inventory; MADRS: Montgomery and Åsberg depression rating scale; YMRS: Young mania rating scale; PANSS: positive and negative symptoms scale; GAF: global assessment of functioning; LIFE: longitudinal interval follow-up evaluation; N/Y: No/Yes; IMI: imipramine dosage equivalent; CPZ: chlorpromazine dosage equivalent; BD: bipolar disorder; SZ: schizophrenia.  Significant group differences are in bold.  ^ Age and sex were included as covariates  ^a^Antidepressant dosage = Imipramine equivalent; ^b^Antipsychotic dosage = Chlorpromazine equivalent.  ^#^ MANCOVA included age, sex and TIV as covariates  ^$^ ANCOVA included age, sex and the indices of ethnicity stratification were added as covariates  **p*<0.05; ** *p* <0.01; *** *p* <0.005 | | | | | | | | | | | | |

| **Supplementary Table 3. Results from two moderation analyses testing either PRS or cognitive group as the moderator of the other’s association with GMV at the peak voxel in the significant left precentral gyrus cluster (MNI coordinates [-28,-10,64]) only in clinical cases whilst accounting for medication.** | | | | | | |
| --- | --- | --- | --- | --- | --- | --- |
|  | *b* | *se* | *t*-statistic | *p*-value | LLCI | ULCI |
|  | | | |  |  |  |
| Direct association with PRS-SZ | |  |  |  |  |  |
|  | **162.570** | **80.228** | **2.026** | **0.046** | **3.275** | **321.865** |
| Direct association with cognitive groups | | |  |  |  |  |
| **CS vs CD** | **0.030** | **0.015** | **2.003** | **0.048** | **0.000** | **0.061** |
|  |  |  |  |  |  |  |
| Interactions: | | | | | | |
| **PRS-SZ x Group (CS vs CD)** | **-188.636** | **53.590** | **-3.520** | **<0.001** | **-295.040** | **-82.232** |
|  |  |  |  |  |  |  |
| PRS-SZ as moderator |  |  |  |  |  |  |
| **Low PRS-SZ CS vs CD** | **0.082** | **0.022** | **3.669** | **<0.001** | **0.038** | **0.127** |
| **Average PRS-SZ CS vs CD** | **0.030** | **0.015** | **2.003** | **0.048** | **0.000** | **0.061** |
| High PRS-SZ CS vs CD | -0.021 | 0.020 | -1.076 | 0.285 | -0.061 | 0.018 |
|  |  |  |  |  |  |  |
| Group as moderator |  |  |  |  |  |  |
| CS | -26.066 | 35.894 | -0.726 | 0.470 | -97.335 | 45.203 |
| **CD** | **-214.702** | **43.404** | **-4.947** | **<0.001** | **-300.882** | **-128.522** |
| PRS-SZ: polygenic risk score for schizophrenia; HC: healthy controls; CS: cognitively spared cases with BD or SSD; CD: cases with BD or SSD with cognitive deficits; se: standard error; LLCI: lower limit 95% confidence interval; ULCI: upper limit 95% confidence interval  Significant associations are in bold.  Medication measures included chlorpromazine equivalent dosage, imipramine equivalent dosage, and mood stabiliser use (yes/no). | | | | | | |


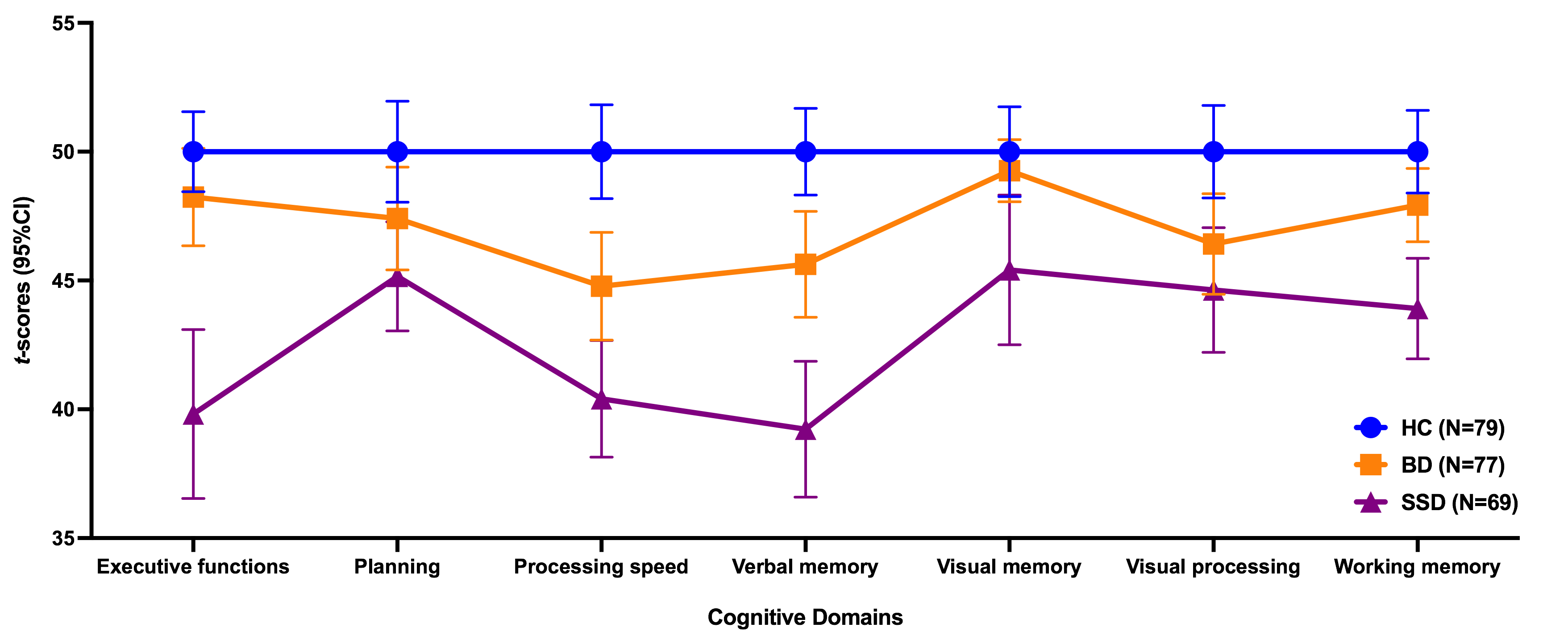


**Supplementary Figure 1. Cognitive profiles of the traditional diagnostic and healthy participant groups**

Compared to the HC group (HC, blue spheres), the schizophrenia spectrum disorders (SSD, purple triangles) group performed at a lower level on all domains, with the bipolar disorder (BD, orange squares) showing intermediate performance.

**
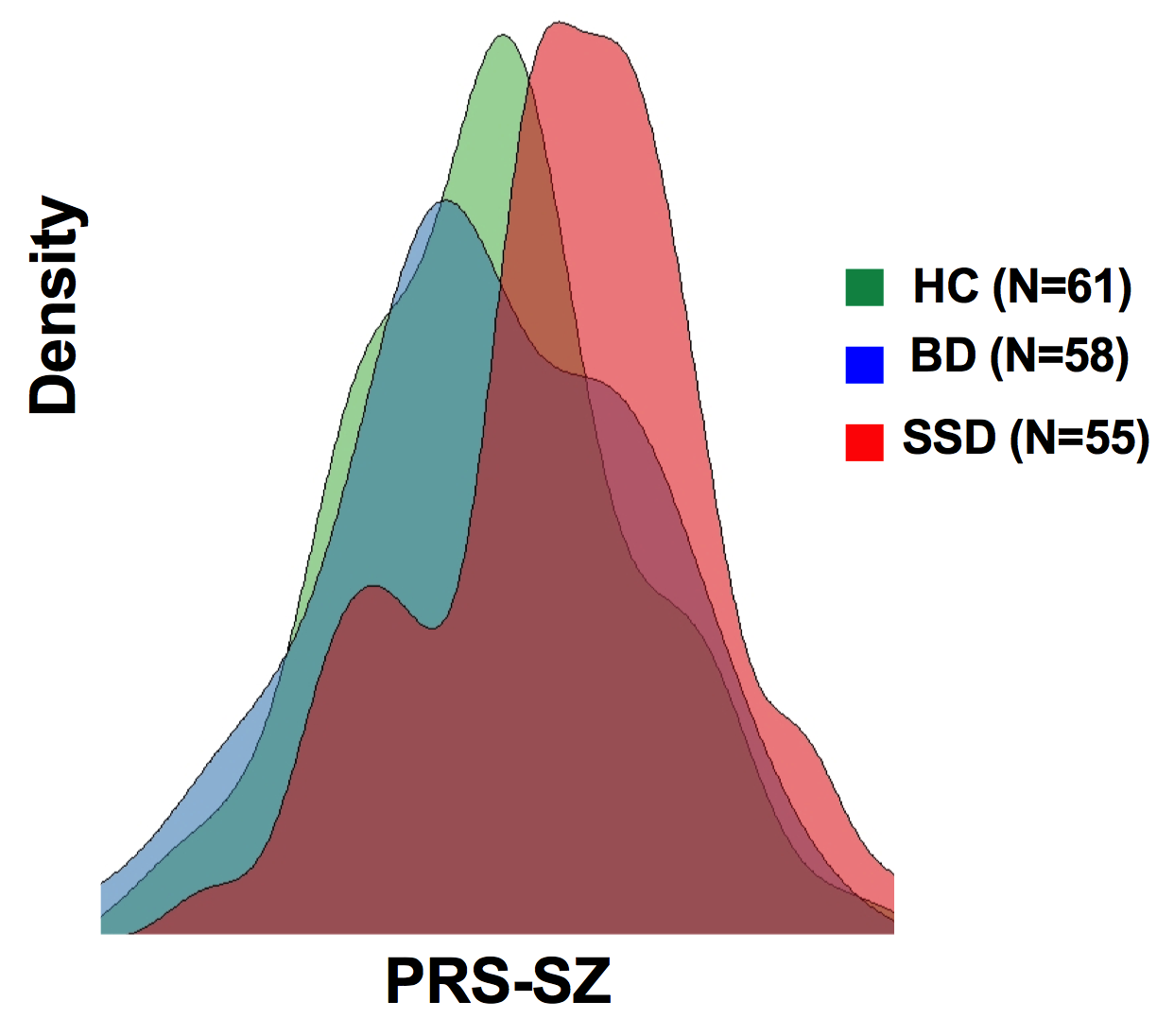
**

**Supplementary Figure 2. Distribution of the polygenic risk scores for schizophrenia (PRS-SZ) among the traditional diagnostic and healthy participant groups.**

The schizophrenia spectrum disorder group (SSD; in red) had significantly higher PRS-SZ than both the group of cases with bipolar disorder (BD; in blue) and the group of healthy controls (HC; in green).
